# Comparing the hierarchy of inter- and intra-species interactions with population dynamics of wine yeast cocultures

**DOI:** 10.1093/femsyr/foad039

**Published:** 2023-09-02

**Authors:** Eléonore Pourcelot, Cleo Conacher, Thérèse Marlin, Florian Bauer, Virginie Galeote, Thibault Nidelet

**Affiliations:** SPO, Univ Montpellier, INRAE, Institut Agro, 34060 Montpellier, France; Department of Viticulture and Oenology, South African Grape and Wine Research Institute, Stellenbosch University, Stellenbosch, 7602, South Africa; Department of Information Science, Centre for Artificial Intelligence Research, Stellenbosch, 7602, South Africa; SPO, Univ Montpellier, INRAE, Institut Agro, 34060 Montpellier, France; Department of Viticulture and Oenology, South African Grape and Wine Research Institute, Stellenbosch University, Stellenbosch, 7602, South Africa; SPO, Univ Montpellier, INRAE, Institut Agro, 34060 Montpellier, France; SPO, Univ Montpellier, INRAE, Institut Agro, 34060 Montpellier, France

**Keywords:** microbial interactions, non-*Saccharomyces*, genetic modification, diversity

## Abstract

In winemaking, the development of new fermentation strategies, such as the use of mixed starter cultures with *Saccharomyces cerevisiae* (Sc) yeast and non-*Saccharomyces* (NS) species, requires a better understanding of how yeasts interact, especially at the beginning of fermentation. Despite the growing knowledge on interactions between Sc and NS, few data are available on the interactions between different species of NS. It is furthermore still unclear whether interactions are primarily driven by generic differences between yeast species or whether individual strains are the evolutionarily relevant unit for biotic interactions. This study aimed at acquiring knowledge of the relevance of species and strain in the population dynamics of cocultures between five yeast species: *Hanseniaspora uvarum, Lachancea thermotolerans, Starmerella bacillaris, Torulaspora delbrueckii* and Sc. We performed cocultures between 15 strains in synthetic grape must and monitored growth in microplates. Both positive and negative interactions were identified. Based on an interaction index, our results showed that the population dynamics seemed mainly driven by the two species involved. Strain level was more relevant in modulating the strength of the interactions. This study provides fundamental insights into the microbial dynamics in early fermentation and contribute to the understanding of more complex consortia encompassing multiple yeasts trains.

## Teaser

Negative and positive changes in population dynamics of wine yeast were determined more at the species level rather than the strain.

## Introduction

Wine is the result of the fermentation of grape must by a variety of microorganisms. Wine yeast species show a great diversity, especially at the beginning of fermentation, including species belonging to the genera *Hanseniaspora, Metschnikowia, Aureobasidium* (yeast-like), *Pichia, Starmerella, Torulaspora, Zygosaccharomyces, Rhodotorula*, and others (Fleet 2003, 2007, Drumonde-Neves et al. 2021). *Saccharomyces cerevisiae*, if detected, is only present at relatively low cell densities in the initial must, but increases during fermentation and is the main species responsible for the completion of the fermentation. Indeed, non-*Saccharomyces* (NS) yeasts, sometimes after an initial rapid growth, tend to decrease during the latter stages of fermentation. This decrease has been attributed to several abiotic factors such as decrease in oxygen, increase in toxic metabolites, including ethanol. More recently biotic factors related to competitive interaction with other species have been highlighted as significant causes for these changes (Fleet 2003). During winemaking, must is often inoculated with *S. cerevisiae* to ensure completion of the fermentation process, however, this is associated with standardization of the final product (Ciani et al. 2010). Thus, there is an increasing interest in introducing non-*Saccharomyces* (NS) to improve the quality of the product and meet consumers' expectations related to more natural products (Galati et al. 2019). Indeed, NS secrete a broader spectrum of enzymes that might release aroma precursors from grapes and have diverse metabolic pathways that allow for variable production of secondary metabolites (Jolly et al. 2006, Polizzotto et al. 2016, Varela and Borneman 2017). However, most of the time, NS alone are not able to ferment to dryness, which has led to the development of mixed starters including *S. cerevisiae* with NS such as *Torulaspora delbrueckii, Metschnikowia fructicola, Lachancea thermotolerans* (Binati et al. 2020). Several NS starters are already available on the market, which are advertised to increase wine aroma complexity, reduce ethanol, or have bioprotection properties to name a few (Roudil et al. 2020). For instance, Renault et al. (2015) showed that co-inoculation of *S. cerevisiae* with *T. delbrueckii* increases the acetate ester content. *Torulaspora delbrueckii* and *Metschnikowia pulcherrima* are also used for bioprotection (Simonin et al. 2018, Sipiczki 2020). *Lachancea thermotolerans* is mainly investigated for its potential for lactic acid production (Morata et al. 2018) and *Starmerella bacillaris* is related to increased glycerol production, which improves mouthfeel (Englezos et al. 2017, Binati et al. 2020). Cocultures of *S. cerevisiae* with *Hanseniaspora uvarum* were related to overyielding of glycerol indicating positive interactions for this functional trait (Harlé et al. 2020).

However, there is limited understanding of how desirable properties emerge from application of multispecies starters, especially regarding the contribution of yeast-yeast interactions. Therefore, to manage fermentations using mixed starters, we need to better understand yeast interactions that might influence the process and final product. Interactions between yeasts can be positive, neutral or negative. Besides the deleterious effect of toxic compounds such as ethanol produced during fermentation, several mechanisms might explain yeast interactions in must (Ciani et al. 2016, Rossouw et al. 2018, Conacher et al. 2019, Bordet et al. 2020). For instance, some *S. cerevisiae* strains, as well as various other yeast species, can produce killer toxins, inhibiting cells either from other species or from the same species (Boynton 2019). *S. cerevisiae* also seems to induce cell death of *L. thermotolerans* and *S. bacillaris* through contact dependent interactions (Englezos et al. 2017, Petitgonnet et al. 2019, Luyt et al. 2021). Rossouw et al. (2015, 2018) also showed that changes in adhesion properties of *S. cerevisiae* significantly affected the survival of other species. Presence of other yeast species have also been found to cause changes in gene expression, for instance *S. cerevisiae* tends to promote genes related to glycolysis and aerobic respiration when in presence of *T. delbrueckii* or *M. pulcherrima* (Tronchoni et al. 2017, Mencher et al. 2021), which might increase its nutrient uptake. Then, the different yeast species could also have overlapping nutritional requirements leading to competition for nutrients such as amino-acids or vitamins (Rollero et al. 2018, Evers et al. 2021). The evidence suggests that there are indeed species-specific yeast-yeast interactions. However, the strain choice could also be an important parameter. Indeed, besides interspecific species diversity, yeasts also show a great intraspecific genetic diversity driven by geographic origin [e.g. *S. bacillaris* (Masneuf-Pomarede et al. 2015)] or the technological origin [e.g *S. cerevisiae* or *L. thermotolerans* (Legras et al. 2007, Hranilovic et al. 2017)]. This genetic diversity is also associated with phenotypic variability between strains isolated from different environments. For instance, *S. cerevisiae* strains from different environments showed different fermentation performances (Camarasa et al. 2011) and competitive abilities (Pérez-Torrado et al. 2018). Even isolates from winery environments display variability in their phenotypic properties such as ethanol resistance, β-glucosidase activity, hydrogen sulphide production, and lactate production (Hranilovic et al. 2017, Morata et al. 2018, Silva-Sousa et al. 2022). This intraspecific diversity may cause variability in chemical composition of fermentation both in monoculture (Bordet et al. 2021) and in coculture (Wang et al. 2016) and therefore impact yeast-yeast interactions and ultimately, the final wine product.

However, it is still unclear which taxonomic level most influences the nature of microbial interactions, or, in other words, is it important to study strain-strain interactions, or, are interaction mechanisms generalized at the species-species level? This gap in understanding can largely be attributed to challenges in differentiating different strains of microorganisms in nature, since most known methodologies used in survey studies cannot distinguish different strains. Thus, the aim of this study was to investigate in simplified systems the determining level of interaction in five yeast species: *H. uvarum, L. thermotolerans, S. bacillaris*, and *T. delbrueckii*, and *S. cerevisiae*. To achieve this, a flow cytometric methodology was developed to distinguish different strains of yeast within mixed cultures, and a high-throughput methodology was used to quantify the population dynamics of all possible pairwise cocultures between 15 strains including 3 strains for the 5 species. This study has applied a versatile methodology for inter-strain interactions and has contributed to the understanding of taxonomic influence on yeast-yeast interactions, using wine yeast as a model.

## Methods

### Strains and medium

In this study, five wine yeast species were used: Saccharomyces cerevisiae and four non-Saccharomyces (NS): Hanseniaspora uvarum, Lachancea thermotolerans, Starmerella bacillaris, and Torulaspora delbrueckii. For each species, three strains were included, all isolated from wine-related environments. All the 15 strains were fluorescently tagged by integrating a fluorescent protein gene into the genome to ensure a better stability of the signal. Origin of each strain can be found in Table 1. Strains were kept at −80°C in yeast peptone dextrose YPD (Peptone; 20 g/L, Glucose 20 g/L; Yeast extract 10 g/L—Sigma-Aldrich, Johannesburg, South Africa) supplemented with 20% of glycerol before being streaked on Wallerstein (WL) nutrient agar (Sigma Aldrich, Darmstadt, Germany).

**Table 1.** List of yeast strains used in this study.

Species	Strain name	Designation	Genotype	Origin	Reference/Provider
***S. cerevisiae***	M2ONO800_1A	Sc1152		France	Marsit et al. 2015
	M2ONO800_1A G2		*TDH3-GFP KANMX*		This study
	59A	Sc59A	*MATa ho AMN1::kanMX4*	France	Ambroset et al. 2011
	59A GFP		*MATa ho AMN1::TEF2Pr-GFP-ADH1-NATMX4*		Marsit et al. 2015
	VIN13	ScVIN13		South Africa	SAWGRI
	VIN13 mCherry		*TDH3-MCHERRY KANMX*		Conacher et al. 2020
***H. uvarum***	CLIB3218	Hu3218		France	CIRM
	CLIB3218 G2		*TDH2-GFP KANMX*		This study
	CLIB3221	Hu3221		France	CIRM
	CLIB3221 G5		*TDH2-GFP KANMX*		This study
	CLIB3118	Hu3118		France	CIRM
	CLIB3118 G2		*TDH2-GFP KANMX*		This study
***L. thermotolerans***	CLIB3053	Lt3053		France	CIRM
	CLIB 3053 G6		*KLTH0G15730-GFP NATMX*		This study
	PY V7-21	LtV7-21		France	SPO, unpublished
	PY V7-21 G5		*KLTH0G15730-GFP NATMX*		This study
	Y1240	LtY1240		South Africa	SAWGRI
	Y1240 BFP		*KLTH0G15730-GFP NATMX*		Conacher et al. 2020
***T. delbrueckii***	CLIB3069	Td3069		France	CIRM
	CLIB3069 G2		*TDEL0E04750-GFP NATMX*		This study
	CLIB3337	Td3337		France	CIRM
	CLIB3337 G1		*TDEL0E04750-GFP NATMX*		This study
	LO544	TdLO544		France	CRBO
	LO544 GFP		*TDEL0E04750-GFP NATMX*		Conacher et al. 2020
***S. bacillaris***	CLIB3147	Sb3147		France	CIRM
	CLIB3147 G1		*TDH3-GFP HPHMX*		This study
	CLIB3334	Sb3334		France	CIRM
	CLIB3334 G3		*TDH3-GFP HPHMX*		This study
	PY V8-1	SbV8-1		France	CIRM
	PY V8-1 G1		*TDH3-GFP HPHMX*		This study

Eight *Escherichia coli* DH5α strains (New England Biolabs, Ipswich, MA, USA) carrying plasmids were used for cassette amplification and were propagated in LB broth (Sigma-Aldrich, St-Louis, USA) supplemented with 100 µg/mL of ampicillin (Sigma-Aldrich).

The high-throughput microplate growth assay were performed using synthetic grape must (SGM425) prepared according to Bely et al. (1990), with 100 g/L of glucose, 100 g/L of fructose and 425 mg/L of yeast assimilable nitrogen (as a mix of ammonium chloride and amino acids).

### Generation of fluorescently tagged yeast strains

For the yeast strains transformed in this study, EGFP (enhanced green fluorescent protein) was integrated into the genome in fusion to *TDH3* gene (or its orthologue in non-*Saccharomyces* species) using homologous recombination. In *S. cerevisiae, TDH3* promoter is a strong promoter known to be expressed throughout fermentation. Cassettes containing the fluorescent protein and an antibiotic selection marker were amplified from different plasmids listed in Supplementary Table 1.

#### Plasmid construction

Plasmids containing the EGFP and different antibiotic resistance gene or specific homology regions were constructed by Gibson assembly (Gibson et al. 2009) using the NEB Builder HiFi DNA Assembly Master Mix (New England Biolabs) and transformed into *Escherichia coli* DH5ɑ (New England Biolabs) following the manufacturer instructions. EGFP in the pFA6 backbone as well as antibiotic resistance genes were obtained from plasmids ordered from AddGene (#44 900, #44 645, Table S1) (Sheff and Thorn 2004, Lee et al. 2013). Where necessary, homologous sequences of approximately 1 kb were amplified from the target species (*T. delbruecki* CLIB3069, *H. uvarum* CLIB3221, *S. bacillaris* CLIB3147). All plasmids were checked by enzymatic digestion (New England Biolabs). A list of the primers and templates used for the amplification of the different Gibson fragments can be found in Supplementary Table 2.

Plasmid DNA was extracted from 3 mL of overnight *E. coli* LB culture with the NucleoSpin Plasmid extraction kit (Macherey Nagel, Düren, Germany) according to manufacturer instructions. Cassettes used for transformation were amplified with a high fidelity enzyme, either the KAPA HiFi kit (Cape Town, South Africa) or Phusion High-Fidelity DNA polymerase (Thermo Fisher Scientific, Vilnius, Lithuania), using primers specific to each species, as listed in Supplementary Table 3.

#### Lithium acetate transformation

Cells were transformed according to Güldener et al. (1996) with some modifications. Fifty milliliters of fresh culture grown in YPD to OD_600_ = 2 were centrifuged at 4415 g for 5 min. Pellets were washed with 20 mL of Tris 10 mM, pH 7.5 and suspended in 25 mL of lithium acetate 0.1 M in Tris-HCl 10 mM, pH 7.5. Cells were incubated for 40 min at room temperature under gentle shaking. After incubation, cells were pelleted at 430 g for 5 min and suspended in 1,125 mL of lithium acetate 0.1 M in Tris-HCl 10 mM, pH 7.5. One hundred microliters of cells were incubated at room temperature with 10 µL of single stranded DNA carrier (Sigma) and 4 µg of PCR fragment for 10 min. Cells were supplemented with 300 µL of PEG 50% in lithium acetate 0.1 M and incubated again at room temperature for 10 min. Cells were then incubated at 42°C for 15 min. After centrifugation at 430 g for 5 min, supernatant was discarded and replaced with 500 µL of YPD. Cells were allowed to recover overnight, then centrifuged at 430 g for 5 min and suspended in 1 mL of Tris 10 mM, pH 7.5 before being plated and incubated for one week at 28°C.

#### Electroporation

The protocol used for the transformation of cells by electroporation was adapted from Gordon et al. (2019). Briefly, cells were inoculated at OD_600_ = 0.5 in 50 mL YPD and grown to OD_600_ = 2. Cells were pelleted by centrifugation at 4415 g for 5 min and rinsed in 25 mL of water. Incubation in 0.1 M LiOAc in 1X TE and DTT 1 M were done according to Gordon et al. 2019. At the final preparation step, cells were suspended in 1 mL of 1 M sorbitol instead of 250 µL. Electro-competent cells were stored at − 80°C. Before electroporation, cells were thawed at room temperature, then centrifuged for 5 min at 430 g. Supernatant was replaced by fresh sorbitol 1 M and cells were kept on ice. Eighty microliters of cells were electroporated at 1.5 kV, 600 Ω, and 10 µF in 0.2 mm cuvettes using Epporator electropotator (Eppendorf, Hamburg, Germany). After electroporation, 1 mL of YPD/sorbitol mix (50:50) was immediately added to the cells. Cells were transferred to test tubes and incubated overnight at 28°C without shaking. Cells were plated onto YPD supplemented with antibiotics and allowed to grow at 28°C for one week.

#### Clone selection and constructions control

For each yeast strain transformation, 8 clones were selected and streaked on fresh YPD supplemented with antibiotic. *S. cerevisiae* and *H. uvarum* transformants were selected on YPD supplemented with 200 µg/mL of G418 (Sigma-Aldrich), *L. thermotolerans* and *T. delbrueckii* transformants were selected on YPD supplemented with 100 µg/mL of nourseothricin (Jena Bioscience, Jena, Germany), *S. bacillaris* transformants were selected on YPD supplemented with 800 µg/mL of hygromycin (Sigma). The correct integration of the cassette at the *TDH3* locus was verified with two PCR using primers outside the integration site and in the cassette. A list of primers used for verification PCR can be found in Supplementary Table 4. Fluorescence was observed by fluorescent microscopy and flow cytometry. Growth of selected clones was compared to WT in YPD and SGM425 to ensure genetic modification did not influence strain behaviour.

#### Microplate growth

**Figure 1.**
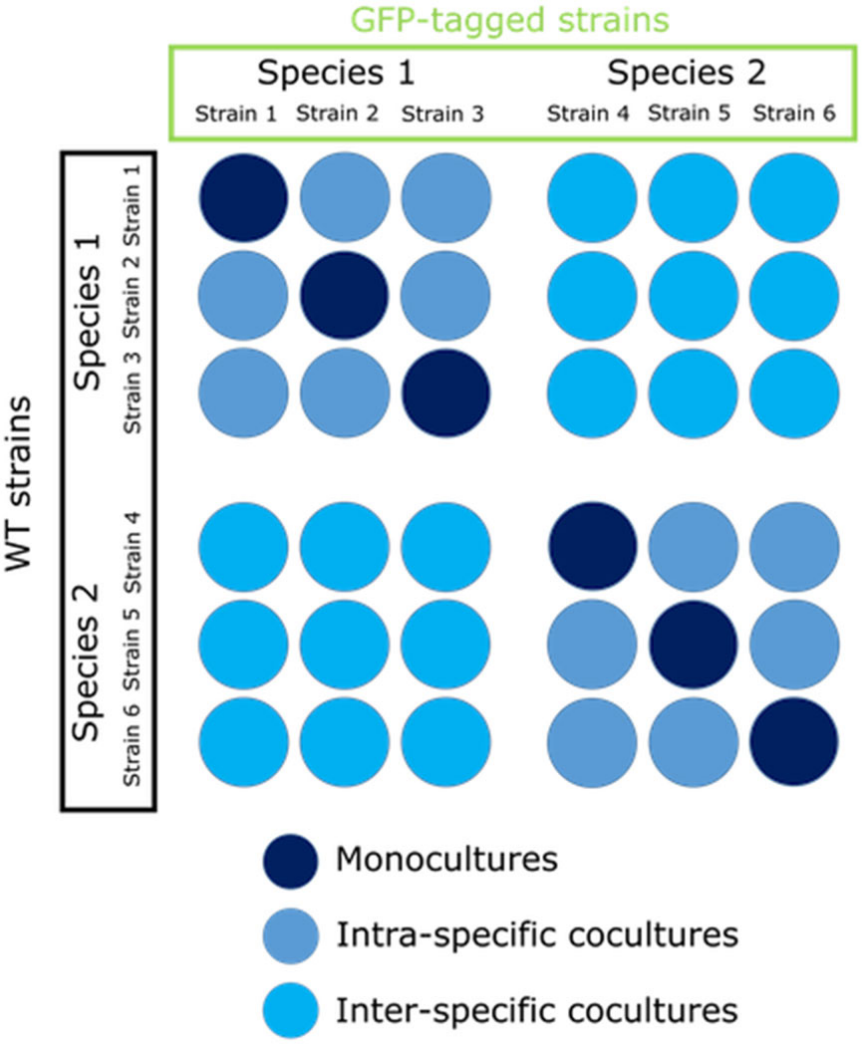
Experimental layout of the mono- and coculture microplate cultures.

A single colony picked from a WL agar plate was propagated in 5 mL YPD for 17 hours at 25°C with shaking at 40 rpm. Then, 100 µL of this preculture was propagated in 5 mL SGM425 for 24 hours at 25°C with shaking at 40 rpm. One mL of culture was harvested, centrifuged, and washed in physiological saline (3000 g—5 min). Cell density and fluorescence of all cells in the culture were measured by cytometry and appropriate volume of culture was used to inoculate SGM425 at 10^6^ cells/mL. Microplates were prepared by mixing 100 µL of SGM425 cell suspension of two strains in each well, as shown on the schematic plan in Fig. 1. Monocultures were inoculated with both the WT strain and its fluorescently tagged counterpart, while cocultures were inoculated with a WT strain and a different fluorescently tagged strain. Plates were covered with a transparent polystyrene lid before being incubated at 28°C for 24 hours with 600 rpm shaking in the Nico plate reader. OD_600_ was measured every 30 min. Despite testing for direct fluorescence reading in microplates, we were unable to measure accurately fluorescence during growth, probably related to media interference. Each coculture was done in biological quadruplicate and monocultures in biological triplicate in at least two independent runs. Initial and final populations of each strain was measured with Cytoflex flow cytometer (Beckman-Coulter, Brea, CA, USA) and fold change was calculated by divided final abundance by the initial abundance. Samples were diluted to include less than 1000 events per second, and acquisition was stopped at 10 000 events. GFP events were detected using the FITC-A channel (λ_ex_=488 nm, filter = 525/40BP), mCherry events with the ECD-A channel (λ_ex_=488 nm, filter = 610/20 BP) and BFP events were detected with the PB450-A channel (λ_ex_=405 nm, filter = 450/45BP). Three different fluorescent channel were used since the study included strains with different fluorescent proteins used in consortia as reported in Conacher et al. (2020).

#### Statistical analysis

Statistical analyses were performed using R software, version 4.2.2 (R Core Team 2022). To obtain the growth rate and area under the curve (AUC) growth parameters, growth curves from the plate reader were analysed with the growthcurver package based on a logistic model (Sprouffske and Wagner 2016). To assess the species and strain effect on growth in monocultures, mixed model analysis were applied to the growth parameters values using the following model:


\begin{eqnarray*}
{Y}_i = \alpha \ + \beta Specie{s}_i\ + {b}_iStrai{n}_i + {\varepsilon }_i
\end{eqnarray*}

$\alpha $ = intercept,${\mathrm{\ }}{Y}_i$ : growth parameter, $\beta Specie{s}_i$ fixed term related to species, ${b}_iStrai{n}_i$ term related to the strain effect, since species and strains constitute hierarchical variables. The model was tested with the *lmer* function (lmerTest package, Kuznetsova et al. 2017). For the growth rate, AUC and maximum population in monocultures, since we observed a strong species effect, differences between strains were tested for each species by ANOVA using the agricolae package (Mendiburu and Yaseen 2020). For the latency, no species effect was observed thus all 15 strains were compared by ANOVA. For cocultures, an interaction index was calculated using the values of both monocultures using the following formula applied to the AUC example:


\begin{eqnarray*}
{\mathrm{Interaction\ index\ for\ AUC}}\ = \ \frac{{AU{C}_{coculture}\ - \ \frac{{AU{C}_{S1}\ + \ AU{C}_{S2}}}{2}}}{{\frac{{AU{C}_{S1}\ + \ AU{C}_{S2}}}{2}}}
\end{eqnarray*}

AUC_coculture_ is the value of the AUC for the coculture of strain S1 and S2, AUC_S1_ and AUC_S2_ are the value of the AUC for the monocultures of strain of S1 and strain S2 respectively. Clustering analyses on the interaction index were performed using the hclust function from the stats (R Core Team 2022) package and clusters were verified by bootstrapping done with clusterboot package (using ‘subset’ and ‘complete’ method, on 1000 bootstrap iteration) function from the fpc package (Hennig 2010). Heatmaps were built following the clustering and interaction index with the ComplexHeatmap package (Gu 2022).

## Results

In this work, we studied the population dynamics in cocultures between 15 different strains from five yeast species: *Saccharomyces cerevisiae, Lachancea thermotolerans, Torulaspora delbrueckii, Starmerella bacillaris, and Hanseniaspora uvarum*. For this purpose, we first generated all strains with a fluorescent tag to measure the population relative abundance of each strain in the cocultures. We then tested all monocultures (coculture consisting of the wild-type strain and its genetically modified counterpart, which carries a fluorescent protein) and pairwise cocultures in synthetic must, following the OD_600_ in microplates (Fig. 1) to enable testing numerous combinations.

### Construction of fluorescently tagged strains

We successfully managed to integrate the cassette containing the EGFP gene at the locus in the five species we studied. However, protocol has been adapted for each species, which should inform future applicability of this methodology for other target non-conventional yeast. For example, LiAc transformation method did not result in transformants for *L. thermotolerans*. Thus, only *S. cerevisiae* was transformed with the LiAc method, whereas electroporation method was applied for all the non-*Saccharomyces* (NS) strains since it is reported to be more effective (Lin-Cereghino et al. 2005, Gordon et al. 2019). Concerning the homologous recombination, in *S. cerevisiae* and *L. thermotolerans*, short homology arms (60 bp) flanking the cassette were sufficient to obtain transformants with the cassette integrated at the locus. Therefore, for *S. cerevisiae* and *L. thermotolerans*, cassettes were amplified from non-specific plasmids using primers containing overhang with homologous sequence to the target integration site (stop codon of the *TDH3* gene) of 60 bp (Table S3). However, for *T. delbrueckii* as well as *H. uvarum*, short homologous sequences were not sufficient to obtain targeted integration, which may be explained by a predominance of the non-homologous-end-joining (NHEJ) DNA repair mechanism in some species (Cai et al. 2019, Navarrete and L. Martínez 2020). Consequently, we used homologous sequences of 1 kb upstream and downstream the target integration site to promote homologous recombination for *T. delbrueckii, H. uvarum* and *S. bacillaris* (Nambu-Nishida et al. 2017, Badura et al. 2021). This required the construction of specific plasmid containing the homologous sequences flanking the cassette with the EGFP and antibiotic resistance genes (Table S1). Every coculture, as well as monoculture, was then constituted of one tagged strain (or clone for monoculture) with one untagged strain (or clone) to enable their discrimination.

### Inter- and intra-specific variability in monocultures

Growth of cocultures and corresponding monocultures were assessed through four kinetics parameters, namely latency time (**Latency** = time in hours for the OD_600_ to exceed 0.25), intrinsic growth rate (**r**), maximum observed OD_600_ (**maxOD**) and the area under the curve (**AUC**). Growth rate (**r**), and area under the curve (**AUC**) were obtained by fitting growth data with a logistic model. The **AUC** is a convenient metric to study microbial growth since it includes all previous metrics (Sprouffske and Wagner 2016, Piccardi et al. 2019). **MaxOD** and **Latency** were directly measured. Growth parameters were assessed after 24 hours of growth since population then reached signal saturation. Moreover, preliminary test did not show significant difference in maximum population after 30 hours compared to 24 hours of growth (data not shown).

**Figure 2.**
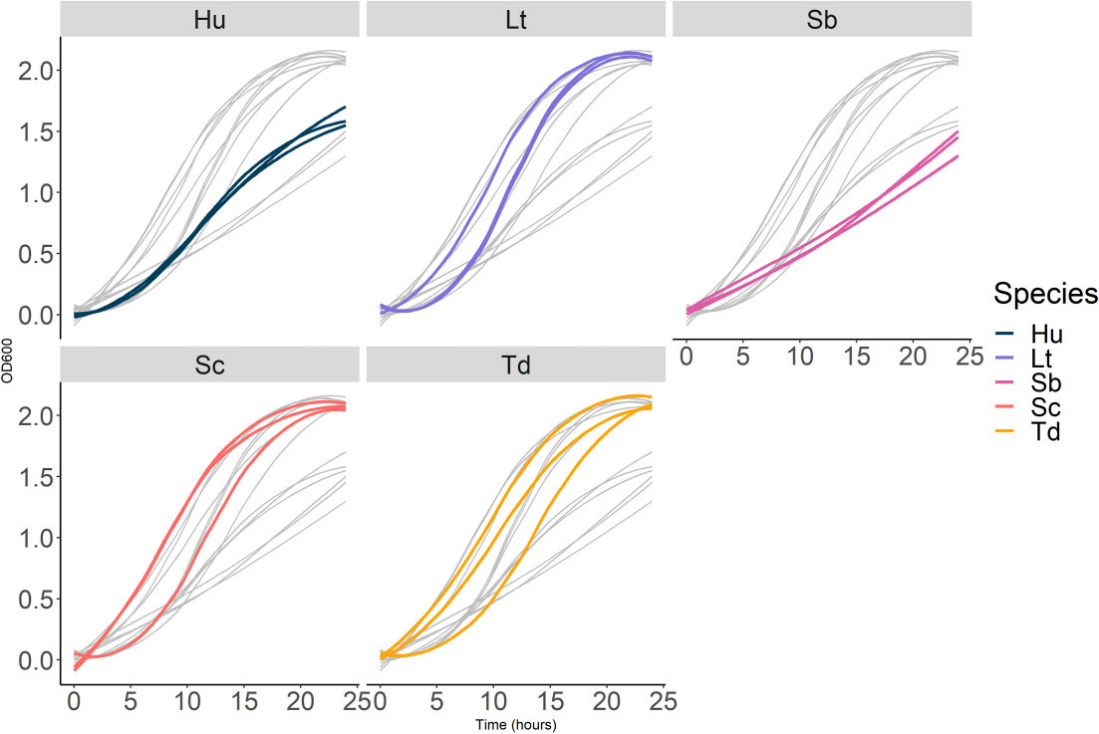
Growth of the 15 monocultures (composed of 50% of fluorescently tagged cells and 50% of WT cells of the same strain) for the 5 species tested in this study: H. uvarum (Hu), L. thermotolerans (Lt), S. bacillaris (Sb), S. cerevisiae (Sc) and T. delbrueckii (Td). All growth curves are represented in grey, growth curves of all three strains of a species are represented in colored lines and species are separated in facets. Monocultures were done in biological triplicates. Curves were ploted using the Loess smoothing method from R tidyverse package.

**Figure 3.**
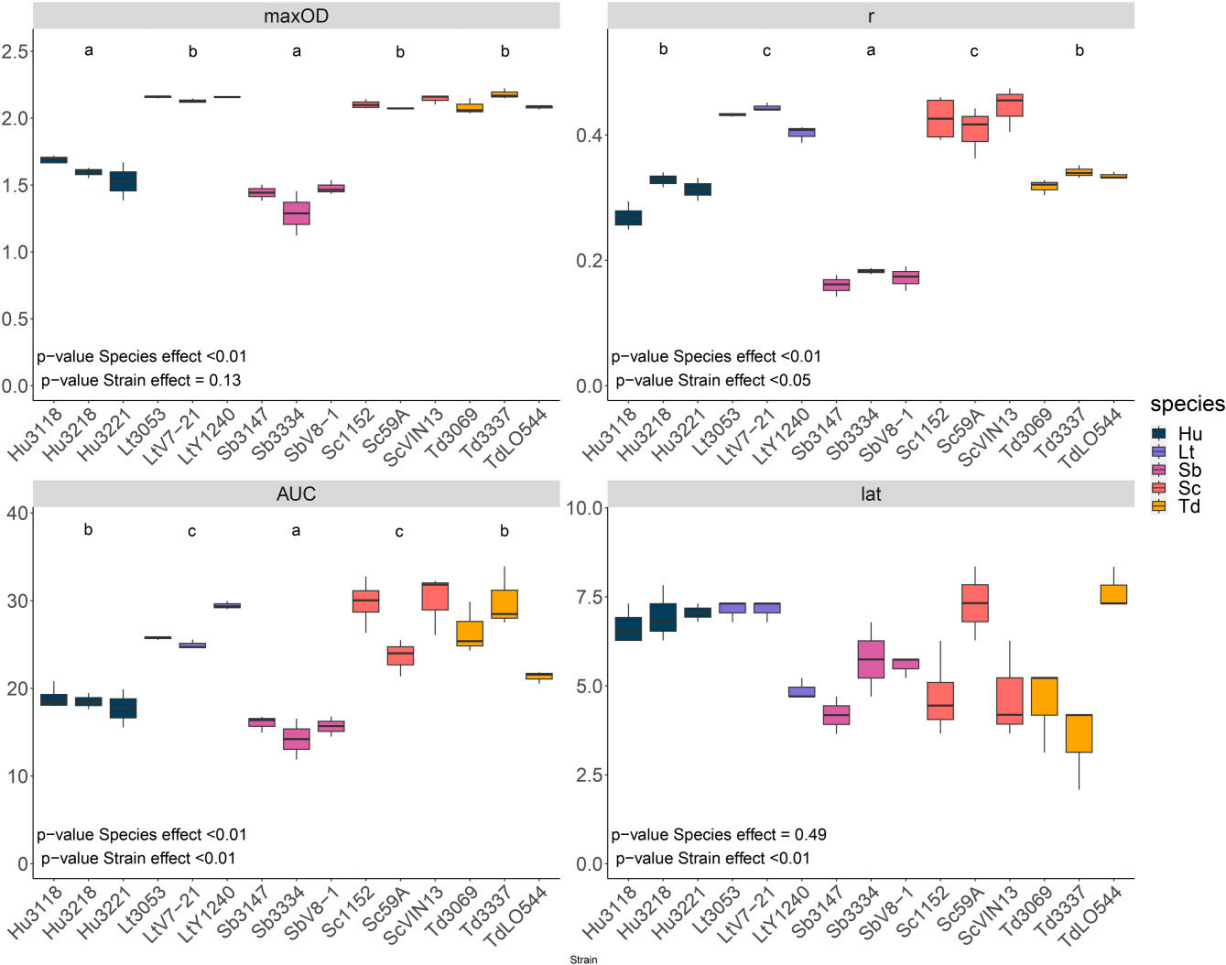
Growth parameters of the 15 monocultures: observed maximum population (maxOD), growth rate hours^−1^ (r), area under the curve (AUC) and latency in hours (lat, time to reach OD600 = 0.25). r and AUC were computed using modelling from the GrowthCurver R package. Monocultures were done in biological triplicates. Letters a,b,c indicate the statistical group of the species. Species effect and strain effect were evaluated using a nested ANOVA: *P*-values of both fixed (species effect) and random effect (strain effect) are indicated for each growth parameter. H. uvarum (Hu), L. thermotolerans (Lt), S. bacillaris (Sb), S. cerevisiae (Sc) and T. delbrueckii (Td).

We first analysed the monocultures and observed a great interspecific variability for growth dynamics (Fig. 2). All studied parameters except **Latency** showed a significant effect between the species (p.value_maxOD_ << 0.01; p.value_r_ << 0.01; p.value_AUC_ < 0.01; Fig. 3). For the **AUC**, two groups of species could be distinguished: *L. thermotolerans, S. cerevisiae* and *T. delbrueckii* showing a higher overall growth (**AUC_Lt_** = 26.8 ± 0.7 h; **AUC_Sc_** = 28.2 ± 1.3 h; and **AUC_Td_** = 26.1 ± 1.5 h) compared to *H. uvarum* and *S. bacillaris* that had lower growth (**AUC_Hu_** = 18.7 ± 0.5 h and **AUC_Sb_** = 15.5 ± 0.6 h; Fig. 3). Strains of the higher growing species *L. thermotolerans, S. cerevisiae, T. delbrueckii* were also logically associated with the highest maximum population (**MaxOD_Td_** = 2.11 ± 0.06, **MaxOD_sc_** = 2.11 ± 0.04, **MaxOD_Lt_** = 2.15 ± 0.02) and higher growth rate (**r_Sc_** = 0.43 ± 0.04 h^−1^, **r_Lt_** = 0.43 ± 0.02 h^−1^) except for *T. delbrueckii*. Indeed, *T. delbrueckii* showed a growth rate (r_Td_ = 0.33 ± 0.01 h^−1^) that is closer to that of *H. uvarum* (**r_Hu_** = 0.30 ± 0.03 h^−1^) that presented a medium maximum population (**MaxOD_Hu_** = 1.62 ± 0.1). Finally, *S. bacillaris* displayed the lowest maximum population (**MaxOD_Sb_** = 1.42 ± 0.13) as well as the lowest growth rate (**r_Sb_** = 0.17 ± 0.02 h^−1^).

Besides interspecific variability, we also observed intraspecific variability for the AUC (*P* value_AUC/Strains_ < 0.01) and **r** (*P* value_r/strains_ = 0.028; Fig. 3). For example, *L. thermotolerans* Y1240 presented an AUC of 29.44 ± 0.46 h, whereas *L. thermotolerans* CLIB3053 and V7-21 presented an AUC of 25.73 ± 0.17 h, 24.97 ± 0.51 h respectively. This difference might be explained by the lower growth rate of *L. thermotolerans* Y1240 strain (r = 0.40 ± 0.01 h^−1^) compared to the two other strains (r_Lt3053_ = 0.43 ± 0.003 h^−1^, r_LtV7-21_ = 0.44 ± 0.01 h^−1^; Fig. 2). Interestingly *L. thermotolerans* Y1240 originated from South Africa while the other two originated from France. Similarly, the strain *T. delbrueckii* LO544, which originates from a different French region, had a 25% lower AUC than the other *T. delbrueckii* strains.

While there is no significant effect of the species for Latency, there is a significant strain effect (p.value_Latency/strains_ << 0.001, Fig. 3). Here the separation is not structured by species but by strains. For example, *L. thermotolerans, S. cerevisiae, T. delbrueckii* and *S. bacillaris* all presented one strain whose latency value was different from the others (Fig. 3). For the 15 strains, the latency ranged from 3.48 ± 1.21 h for Td 3337 to 7.66 ± 0.59 h for Td LO544 with an average of 5.98 ± 1.51 h.

### Evaluating species-species and strain-strain interaction in cocultures

**Figure 4.**
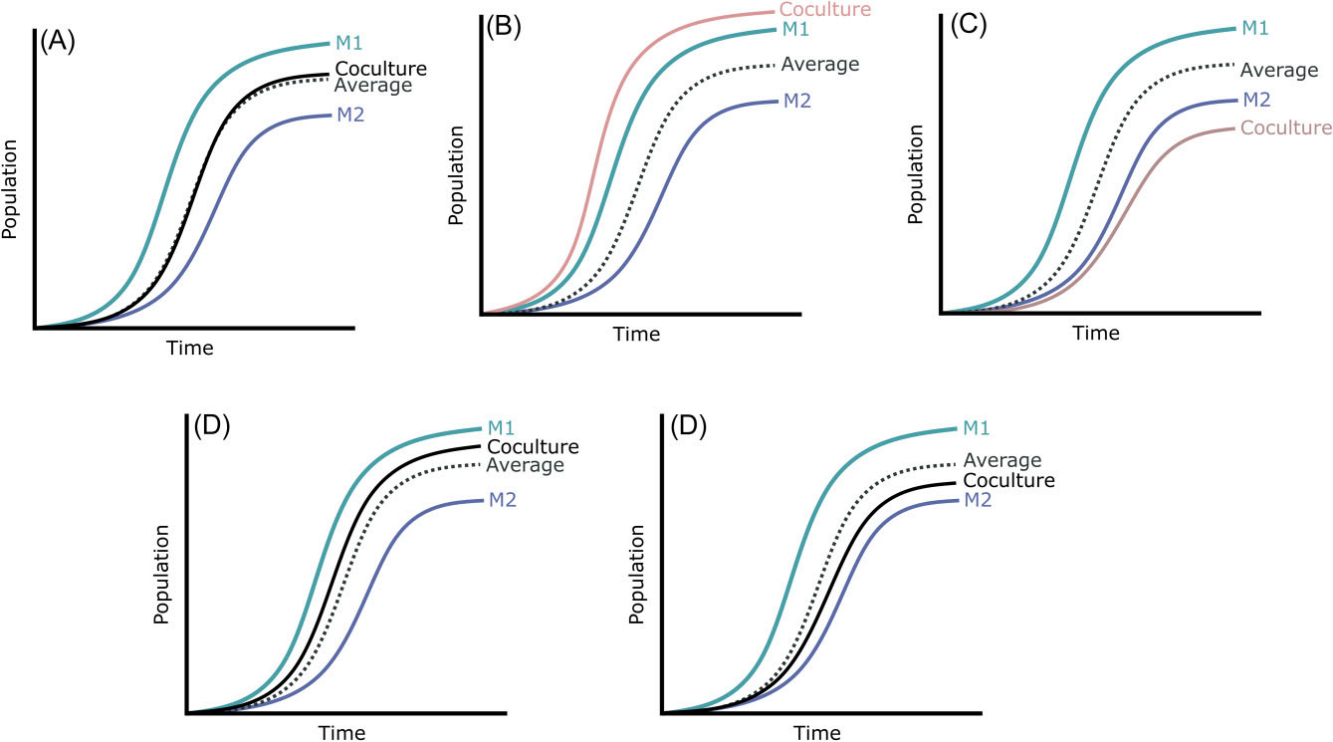
Classification of the possible outcomes of co-cultures compared to monocultures. A: No interaction—co-culture relates to the average of both monocultures, B: Overyielding—coculture is greater than both monocultures, C: Underyielding—co-culture is worse than both monocultures, D: Little interaction—coculture significantly differs from the average of both monocultures but remains in the range of both. S1 = strain 1, S2 = S2, in dashed line = the calculated average of both monocultures.

To determine how the species and strain effect affected growth in cocultures, we tested whether there was a significant effect on the four growth parameters in all cocultures. To this end, we distinguished three types of culture: S_1_ strain 1 monoculture, S_2_ strain 2 monoculture and Co the corresponding coculture comprising strain 1 and strain 2. Theoretically, four typical outcomes can be distinguished (Fig. 4): when the T-test did not show significant difference between the parameter value of the coculture and the average of the parameter value of the monocultures, it was called case A [for example: AUC_Co_ ≈ (AUC_S1_ + AUC_S2_)/2], and there is no perceived change in dynamics. If the coculture growth parameter is statistically superior to the best monoculture or inferior to the worst monoculture, it was respectively called cases B and C, where there is a clear positive or negative interaction. In all other cases (case D) the parameter value of the coculture is between monoculture parameters values but it is not possible to determine if there is an interaction or only the effect of respective population density (Fig. 4D). In addition to classifying results according to these categories, we also calculated an interaction index (Id) by comparing for each parameter (growth rate, maximum population, latency and AUC) the coculture value to both monoculture values. We used this index to perform a clustering analysis of all cultures.

**Figure 5.**
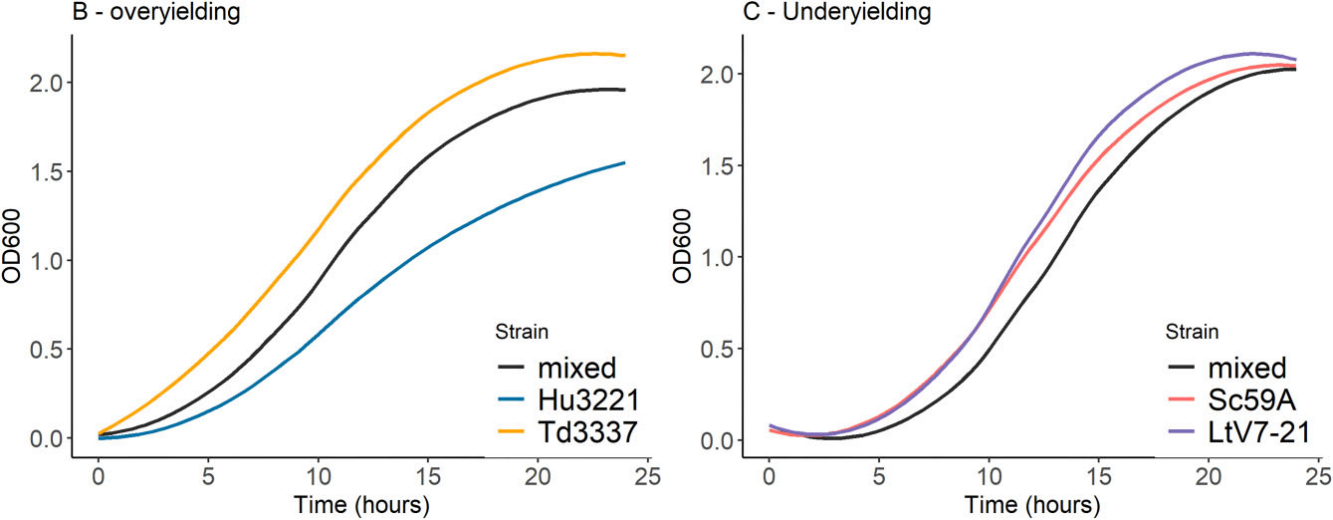
Examples of case B (left panel) and C (right panel). The only overyielding observed was for the growth rate of the coculture of H. uvarum 3221 and T. delbrueckii 3337. Underyielding was observed with the maximum population of the coculture of L. thermotolerans V7-21 and S. cerevisiae 59A. Four biological replicates were run for each cocultures.

When focusing on the AUC, a great majority of cocultures were in the A (77%) and D (33%) cases (Fig. 4, Table 2) where there is little to no perceived interactions. For the other parameters, most cocultures were also in case A or D (Table 2). A few underyielding cases were identified for the maximum population (5%) such as in the coculture of *S. cerevisiae* 59A with *L. thermotolerans* V7-21 (Fig. 5). Only one overyielding case (B) was found for the growth rate in the cocultures of *H. uvarum* 3221 with *T. delbrueckii* 3337 respectively (Fig. 5). Overall, these results would suggest that the measured growth parameters did not appear to identify strong interactions in cocultures.

**Table 2.** Count and percentage of cocultures types for all four growth parameters. A: no statistical difference between the coculture parameter value and the average of both monocultures (evaluated by T-test). B: overyielding—the coculture parameter value is higher than the maximum of both monocultures (T-test with maximum). C: underyielding—the coculture parameter value is lower than the minimum of both monocultures (T-test with minimum). D: the coculture parameter value is between both monocultures and statistically different from the average of both monocultures.

Growth parameter	Coculture type	Number of cases	Percentage of cases
***Area under the curve (AUC)***	A	81	77
	D	24	23
***Latency (lat)***	A	69	66
	B	2	2
	D	34	32
***Maximum population (maxOD)***	A	60	57
	C	5	5
	D	40	38
***Growth rate (r)***	A	73	70
	B	1	1
	C	2	2
	D	29	28

**Figure 6.**
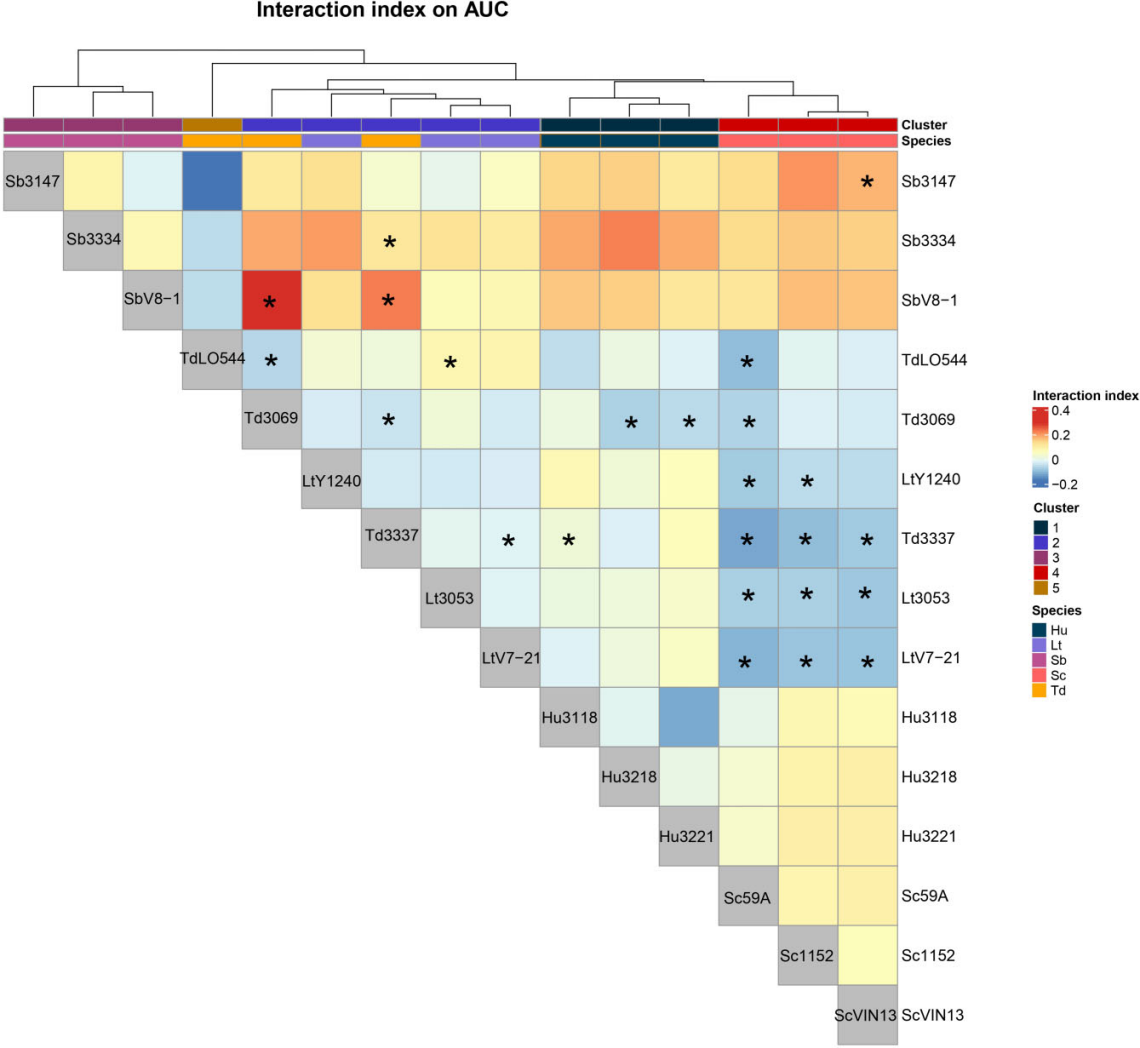
Heatmap of the AUC index: positive index means an overall higher growth of the coculture. * denotes cocultures whose AUC is significantly different from the average AUC of both monocultures. Colors of cluster and species, as well as addition of the * were edited manually from the PDF file. The original figure from R is available in the data repository.

As we did not observe many extreme changes in population dynamics such as over- and under-yielding, we thus used an interaction index for all four growth parameters to assess the strength of the interaction in addition to the quality of the interaction. Noteworthy, for positive interactions, it was not possible to determine whether the interaction was positive for both species or only one. Regarding the AUC-based heatmap (Fig. 6), negative interaction were seen in cocultures of all strains of *S. cerevisiae* with *T. delbrueckii* CLIB3337 and *L. thermotolerans* V7-21. The strain *S. cerevisiae* 59A also showed significant negative interactions with all *T. delbrueckii* strains and all *L. thermotolerans* strains. On the contrary, cocultures of *S. bacillaris* with *S. cerevisiae, H. uvarum* or *T. delbrueckii* tended to have positive index but only cocultures of *S. bacillaris* CLIB3147 with *S. cerevisiae* VIN13 or *S. bacillaris* with either *T. delbrueckii* CLIB3337 or *T. delbrueckii* CLIB3069 showed statistically significant positive interactions.

The other growth parameters also corresponded to the interactions quantified in terms of AUC. Cocultures of *S. bacillaris* with other species tended to be positive. However, contrary to the other parameters, negative interactions were revealed by the latency parameter in cocultures of *S. bacillaris* with *L. thermotolerans or T. delbrueckii* with even underyielding for *S. bacillaris* 3147 and *L. thermotolerans* Y1240 (Fig. S2). For the latency, a positive interaction index indicates a longer latency time, hence a delayed growth so a negative interaction.

Negative interactions between *S. cerevisiae* and *T. delbrueckii or L. thermotolerans* were also revealed by the interaction index of growth rate, maximum population, and latency. Underyielding (Fig. 3C) was observed for growth rate in cocultures of *S. cerevisiae* VIN13 with *L. thermotolerans* CLIB3053 or *L. thermotolerans* V7-21 as well as for the maximum population in cocultures of *S. cerevisiae* 59A with *T. delbrueckii* 3069, *T. delbrueckii* LO544 and *L. thermotolerans* V7-21 (Fig. S3). The latency also revealed some negative interactions between *L. thermotolerans* and *T. delbrueckii*.

In addition to interspecific interactions, the maximum population parameters revealed intra-specific negative interactions for the cocultures of *T. delbrueckii* 3069 with *T. delbrueckii* LO544, and *L. thermotolerans* CLIB3053 with *L. thermotolerans* Y1240 with negative interaction strong enough to induce underyielding (Fig. S2).

### Analysis of the interaction matrices

Another interesting result obtained from the heatmap is the structure of the interaction matrix. The clustering of rows and columns was based only on similarity of the interaction index between strains (Euclidean distance with complete linkage), and the relevance of clusters was checked by bootstrapping. We observed that the resulting clusters based on AUC interaction index values fit species level for *H. uvarum, S. cerevisiae* and *S. bacillaris* (Fig. 6). Cluster 2 includes both *L. thermotolerans* strains and *T. delbrueckii* strains (CLIB 3069 and CLIB 3337). Cluster 5 includes *T. delbrueckii* LO544 only, however bootstrapping analysis of the clusters showed the significance of cluster 5 was low (Jaccard index = 0.64). Altogether, this would suggest that species rather than strain is the main level determining interactions in cocultures with two species, even though strain can impact the strength of the interaction since values of the interaction index varied between strains of the same species. For instance, *T. delbrueckii* 3069 and 3337 showed great positive interactions with *S. bacillaris* V8-1 whereas this interaction was more neutral with *S. bacillaris* 3147.

Clustering on the growth rate interaction index also fitted mostly to the species level; with the only discrepancy being *S. cerevisiae 59A* that clustered with *T. delbrueckii* strains (Fig. S1). There were some examples of strain-level interactions on growth rate, for example, *T. delbrueckii* 3069 and 3337 exhibited opposing negative and positive interactions respectively with *H. uvarum*. For the maximum population and latency time, there were differing clustering patterns (Figs S2 and S3). For the interaction index data from the latency time, strains of *S. bacillaris* and strains of *T. delbrueckii* did not group together, while strains of other species did group into respective clusters (Fig. S2). For the maximum population metric, *S. cerevisiae, L. thermotolerans and* two strains of *T. delbrueckii* strains grouped together in a single cluster, while strains of other species did group into respective clusters (Fig. S3).

The fact that structures of the interaction matrix didn't follow the species level for the maximum population may be related to limitations of measures by OD that tend to be quickly saturated (Stevenson et al. 2016). This might also result from the intrinsic growth phenotypes of each strain since monocultures that were already grouped together for the maximum population, namely *S. cerevisiae, T. delbrueckii*, and *L. thermotolerans*, were clustered together. The structure found for the phenotype of monocultures might also explain the fact that in cocultures, as for monocultures, no predominant species effect was observed for the latency.

### Population dynamics highlighted by change in relative abundance

To evaluate the influence of coculture on the population composition, which is an important metric in determining competitive phenotypes, we calculated fold change with the relative abundance of both strains in each cocultures at start (T0) and after 24 hours of growth (T24).

**Figure 7.**
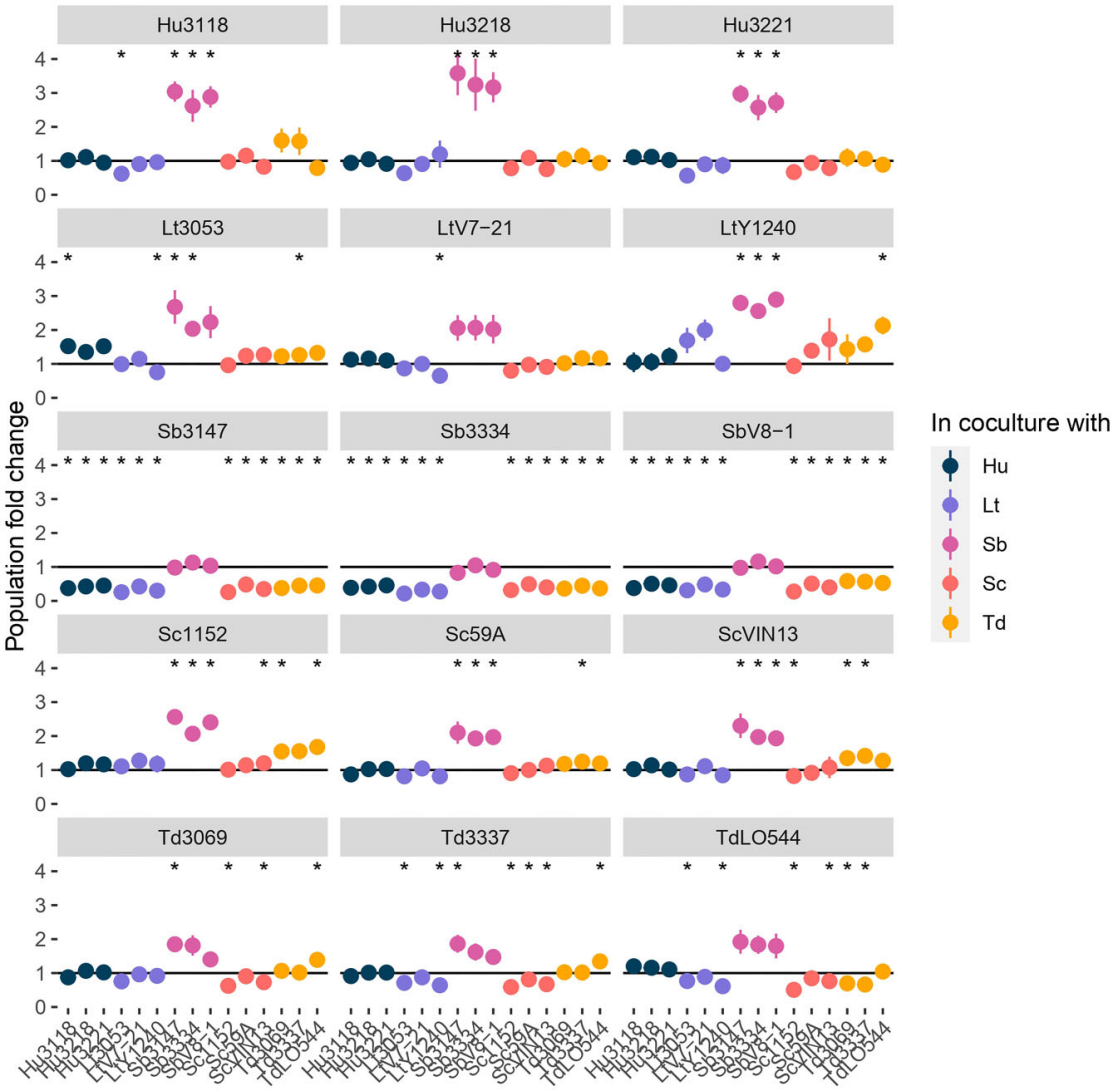
Population fold change after 24-hour growth in SGM425 for each strain (panel) in coculture with the strain indicated in the column. Colors of the dot correspond to the species of the strain in the column. * denotes significant difference of the fold change to 1 as tested by t-test.

The population abundances of most strains within coculture stayed consistent throughout the measured samples. In particular, for monocultures, no change in relative abundance of the wild-type and tagged strains were observed, indicating transformants did not exhibit differences in fitness as compared to wild-type strains (Fig. 7). On the contrary, all strains of *S. bacillaris* showed significant decrease in relative abundance after 24 h when in coculture with other species, with a 2-fold reduction on average for the 3 strains (fold change = 0.52 ± 0.02). This is logically associated with an increased abundance of the counterpart species. The fold change in favour of better growing strains when cocultured with *S. bacillaris* could explain the positive interaction observed with maximum population and AUC for these cocultures (Fig. 6, Fig. S3) since the maximum population observed is the result of their respective maximum OD600. Fold change of cocultures of other species were less sizable and few were significant. Surprisingly, besides with *S. bacillaris, S. cerevisiae* abundance increased only in cocultures with *T. delbrueckii*. For instance, *S. cerevisiae* strain 1152 had a fold change of 1.55 ± 0.09 and 1.68 ± 0.2 when cocultured with *T. delbrueckii* 3069 and LO544 respectively. *L. thermotolerans* had significant increased abundance in some strain-specific cases such as *L. thermotolerans* 3053 with *H. uvarum* 3118 (1.52 ± 0.13) or *L. thermotolerans* Y1240 in coculture with *T. delbrueckii* LO544 (2.13 ± 0.26).

In terms of strain-strain differences, the data shows fold-change variations in intraspecific cocultures of *T. delbrueckii* (Fig. 6). For instance, *T. delbrueckii* LO544 relative abundance declined when in presence of either *T. delbrueckii* 3069 or 3337 (fold change of 0.70 ± 0.06 and 0.66 ± 0.08, respectively). Two factors could explain this intraspecific effect. It could either be related to the longer latency phase of the strain LO544 (Fig. 3). It might also be the results of intraspecific negative interactions between strains of *T. delbrueckii* as observed with the significantly reduced maximum population in cocultures of *T. delbrueckii* LO544 with *T. delbrueckii* 3069 or 3337 compared to monocultures. On the contrary *H. uvarum, S. bacillaris*, and *S. cerevisiae* showed no fold change when cocultured with a strain of the same species.

## Discussion

In the present study, we conducted an experiment aiming to explore ecological questions regarding the relative importance of species and strain for determining the nature and intensity of yeast-yeast interactions. For this purpose, we studied population-specific and over-all growth kinetics of all pairwise cocultures of 15 total strains comprising 5 different yeast with a simplified framework of the wine environment.

The approach used in this study involved tagging all strains with fluorescent proteins to enable species detection with cytometry at the end of growth. With the current lack of information on the genetics of wine related NS, molecular tools available are still scarce and transformation of NS remains highly challenging (Masneuf-Pomarede et al. 2016). To our knowledge, only one article reported transformation with homologous recombination for *H. uvarum* (Badura et al. 2021), and one article reported transformation of *Starmerella bombicola* (Gonçalves et al. 2018) but none specifically reported transformation of *S. bacillaris*. The strains we constructed will therefore be valuable tools in the future for studies on yeast. However, more research should focus on transformation of NS since there are still species that were reported to be unable to integrate cassette at the target locus, such as *M. pulcherrima* (Gordon et al. 2019, Moreno-Beltrán et al. 2021). The tagging strategy coupled with cytometry, which is a powerful analytical tool for population analysis of fermentation (Longin et al. 2017), enabled us to discriminate two strains in cocultures. This approach can also be applied to the detection of more species (Conacher et al. 2020). A limitation of the study is that our data only focus on the growth patterns of species, which limit our understanding on the respective growth of each strain, as well as the impact of strains on the resulting wine composition. Moreover, oxygen availability in microplates do not reflect real fermentation conditions. However, this method makes it possible to monitor growth of multiple pair-combination, and is very useful for high-throughput protocols.

In this study, comparison of growth kinetics of cocultures and monocultures based on an interaction index suggested a predominant effect of the species level over the strain on the interactions structure. The cluster analysis resulting from the interaction matrix for the AUC and the growth rate displayed an overlap of clusters with the species level, despite the initial intra-specific diversity observed in monocultures. Altogether, our results would indicate that population dynamics between two species are mainly driven by the species type, while the strain would mostly affect the strength of the interaction. This is an important consideration in the design of synthetic communities.

It is interesting to note that most cocultures displayed little to no perceived interactions (cases A Table 2, Fig. 3). These results would reflect those observed in some bacterial cocultures, either from strains of the same species or spanning several families and genera, where inhibition interactions constituted less than 15% of pairwise interactions (Russel et al. 2017, Ramia et al. 2020). It could also result from the microtiter plate method that do not allow a detailed analysis of minor changes, especially in the respective growth of each strain. Nevertheless, our study provides a broader insight in yeast interactions, especially NS/NS interactions that are still poorly documented (Zilelidou and Nisiotou 2021). Most interactions that have been studied to date are negative interactions found between *S. cerevisiae* and NS, but some positive interactions through crossfeeding were also identified between *L. thermotolerans* and *Zygosaccharomyces* spp. for example (Csoma et al. 2020). Further research is needed to confirm our findings, especially with more species to include more genera as well as species from the same genera similarly to a recent study that investigated cocultures of 60 strains of wine yeast in coculture with *S. cerevisiae* (Ruiz et al. 2023). In addition, our findings are limited to only one synthetic media, whereas interactions are known to be modulated by environments (Piccardi et al. 2019, Gao et al. 2021). Thus, it would be relevant to test these combinations in environments closer to actual wine fermentation, for instance using different natural grape musts. Indeed, the wine environment includes various stressors that have been shown to influence population dynamics, even at the strain level as shown by Schmidt for *S. cerevisiae* (Schmidt et al. 2020). The importance of strains variability might then lie in the adaptability of one species to different environments. Moreover, the strains evaluated here have all been isolated from wine environments. Evidence clearly supports that this anthropic environment has evolutionarily shaped the associated yeast community (Conacher et al. 2019, De Guidi et al. 2023). The interactions between the species and strains evaluated here might therefore be the result of wine-specific evolutionary adaptations linked to direct interspecies biotic selection pressures. It would be interesting to add strains isolated from other environments to our analysis.

Phylogenetic or metabolic distance might be part of the explanation of the relevance of species in pairwise interactions. For instance, Russel et al. showed that bacterial species phylogenetically closer tended to display higher competition, the assumption being that phylogenetically closer species have closer niches (Russel et al. 2017). Peay et al. (2012) obtained similar results for yeasts in a floral nectar flower community assembly. Our data would be in accordance with these findings since we observed significant negative interactions between species such as *S. cerevisiae, T. delbrueckii* or *L. thermotolerans*, even though Ruiz et al. (2023) identified positive interactions between *S. cerevisiae* and *L. thermotolerans* or *T. delbrueckii*. In our study, these three species showed similar growth patterns in monocultures, except for a lower growth rate for *T. delbrueckii* strains (Fig. 3), and are known to be phylogenetically closer together than *S. bacillaris* and *H. uvarum* (Kurtzman 2011, Lemos Junior et al. 2018). Moreover, they also seem to have similar amino-acid consumption and are reported to be intermediate or good fermentative species, which might result in higher competition (Prior et al. 2019, Roca-Mesa et al. 2020). However, if it was only a question of phylogenetic distance, then there would be very high intraspecific competition which we observed only for *T. delbrueckii* and *L. thermotolerans* (fold change and maximum population; Fig. 7; Fig. S3) while interactions in intraspecific cocultures for the other 3 species were mostly neutral. This might indicate other interaction mechanisms are also involved, such as contact-dependent interactions for example. For instance, *S. cerevisiae* seems to induce contact-dependent cell-death of other species such as *L. thermotolerans* (Petitgonnet et al. 2019, Luyt et al. 2021). Although, for *T. delbrueckii*, Taillandier et al. (2014) excluded contact-mediated interactions between *T. delbrueckii* and *S. cerevisiae* but instead hypothesized that *T. delbrueckii* was sensitive to a killer toxin produced by *S. cerevisiae*. On the other hand, negative interactions mediated by cell-contact have been reported between *S. bacillaris* and *S. cerevisiae*, whereas we mostly identified positive interactions between *S. bacillaris* strains and the other species. However, our data must be interpreted with caution since our method does not allow us to evaluate the respective growth of *S. bacillaris* and *S. cerevisiae*. Thus *S. bacillaris* could be outgrown by other species due to its slow growth seen in monocultures, consistent with its fold change < 1, and reaching a maximum population closer to the other species' monoculture.

In conclusion, the experimental design implemented in this study, based on a comparison of growth in cocultures and monocultures of 15 wine yeast strains including 5 different species, provided insight in the relevance of the species level and strains in population dynamics in cocultures. Our results indicate that the species level would be the driver of the type of interaction, whereas the strain would modulate the intensity of the interaction. This theoretical knowledge offer new perspectives on the interactions between yeast, especially between non-*Saccharomyces* species and raise questions on the different mechanisms involved in inter- and intra-specific interactions.

## Supplementary Material

foad039_Supplemental_File

## Data Availability

All source data and codes, from which the figures are based, are available through the Zenodo repository: https://doi.org/10.5281/zenodo.7915768 under a Creative Commons Attribution (CC-BY) licence.
