# Supplementary material for: Comparing the hierarchy of inter- and intra-species interactions with population dynamics of wine yeast cocultures
Source: FEMS Yeast Res. 2023 Sep 2;23:foad039. doi: 10.1093/femsyr/foad039 (PMC10532119; doi:10.1093/femsyr/foad039)
Supplement: foad039_Supplemental_File [file foad039_supplemental_file.docx]

Supplementary information

**Supplementary Table 1: Plasmid list**

| **Plasmid name** | **Features** | **Purpose** | **Origin** | **AddGeneRef/MTF** |
| --- | --- | --- | --- | --- |
| **pFA6a-link-yoEGFP-Kan** | pFA6 backbone, yeast optimised EGFP, Kanamycin resistance gene | Non-specific tagging with EGFP using G418 selection | Lee et al., 2013 | #44900 |
| **pFA6a-link-yEmCitrine-Hygro** | pFA6 backbone, yeast optimised monomeric Citrine, Hygromycin resistance gene | Construction of plasmid with hygromycin resistance gene | Sheff et al., 2004 | #44645 |
| **pFA6a-TEF2Pr-eGFP-ADH1-NATMX** | pFA6 backbone, EGFP with TEF2 promoter , Nourseothrcin resistance gene | Construction of plasmid with nourseothricin resistance gene | Breslow et al., 2008 | 2559 |
| **pFA6a-link-yEGFP-NAT1** | pFA6 backbone, yeast optimised EGFP, Kanamycin resistance gene | Non-specific tagging with EGFP using nourseothricin selection | This study | 4882 |
| **pFA6a-link-yoEGFP-hph** | pFA6 backbone, yeast optimised EGFP, hygromycin resistance gene | Non-specific tagging with EGFP using hygromycin selection | This study | 4884 |
| **pFA6-TDH3.1kb.Hu-GFP-Kan** | pFA6 backbone, yeast optimised EGFP, Kanamycin resistance gene, homologous sequence of 1 kb specific to *H. uvarum* TDH3 ortholog | *H. uvarum* specific tagging with EGFP using kanamycin selection | This study | 4880 |
| **pFA6-TDH3.1kb.Sb-GFP-hph** | pFA6 backbone, yeast optimised EGFP, Hygromycin resistance gene, homologous sequence of 1 kb specific to *S. bacillaris* TDH3 ortholog | *S. bacillaris* specific tagging with EGFP using hygromycin selection | This study | 4888 |
| **pFA6-TDH3.1kb.Td-GFP-hph** | pFA6 backbone, yeast optimised EGFP, Hygromycin resistance gene, homologous sequence of 1 kb specific to *T. delbrueckii* TDH3 ortholog | *T. delbrueckii* specific tagging with EGFP using hygromycin selection | This study | 4890 |

**Supplementary Table 2: Primer list for plasmid construction**

| **Plasmide name** | **Primer name** | **Sequence (5'→3')** | **Template DNA** |
| --- | --- | --- | --- |
| **pFA6a-EGFP-hphMX** | TSapF_TSap-Hyg | agggcaaaggaataatcagtac**tgacaataaaaagattcttg** | *pFA6a-link-yoEGFP-Kan* |
|  | TSapR_TSap-Hyg | agttcaggctttttacccat**ggttgtttatgttcggatgt** |  |
|  | HygF_Tsap-Hyg | acatccgaacataaacaacc**atgggtaaaaagcctgaact** | *pFA6a-yEmCitrine-Hygro* |
|  | HygR_Tsap-Hyg | agaatctttttattgtcagt**actgattattcctttgccct** |  |
| **pFA6a-EGFP-natMX** | FPF_FP-NAT | tgagcatgccctgcccctaa**tcagtactgacaataaaaaga** | *pFA6a-link-yoEGFP-Kan* |
|  | FPR_FP-NAT | tcgtcaagagtggtacccat**ggttgtttatgttcggatgt** |  |
|  | NATF_FP-NAT.lg | acatccgaacataaacaacc**atgggtaccactcttgacgac** | *pFA6a-TEF2Pr-eGFP-ADH1-NATMX4* |
|  | NATR_FP-NAT.lg | ctttttattgtcagtactga**ttaggggcagggcatgctca** |  |
| **pFA6-TDH3.Hu.1kb-GFP-Kan** | pFA6-TDH3-Hu1kb-For | atttcttaaactttttatat**tgatatcagatccactagtggcct** | pFA6a-link-yoEGFP-Kan |
|  | pFA6-TDH3-Hu1kb-Rev | ccgaaaccgttaatggaaac**gatccgtcgacctgcagcgt** |  |
|  | H1-TDH3-Uni1kb-For | acgctgcaggtcgacggatc**agttgctattaacggtttcgg** | *H. uvarum* CLIB3221 |
|  | H1-TDH3-Hu1kb-Rev | attaaaccagcaccgtcacc**gttcttgaaaacgtgttcga** |  |
|  | HR-ForHu | gataacgaattcggttactccaccagagttgttgacttagtcgaacacgttttcaagaac**ggtgacggtgctggtttaat** | pFA6a-link-yoEGFP-Kan |
|  | HR-RevHu | taatcagttaaaaaaagaaaaaaggacaaatatccttttttagaattatttttgactaat**tcgatgaattcgagctcgtt** |  |
|  | H2-TDH3-Hu1kb-For | aacgagctcgaattcatcga**attagtcaaaaataattctaaaaaagg** | *H. uvarum* CLIB3221 |
|  | H2-TDH3-Hu1kb-Rev | cactagtggatctgatatca**atataaaaagtttaagaaataaaacga** |  |
| **pFA6-TDH3.1kb.Sb-GFP-hph** | pFA6-TDH3-Sb1kb-For | ggagccgacgaggtcacaaa**tgatatcagatccactagtggcct** | pFA6-GFP-hphMX |
|  | pFA6-TDH3-Sb1kb-Rev | ccgaaaccgttaataccaac**gatccgtcgacctgcagcgt** |  |
|  | H1-TDH3-Uni1kb-For | acgctgcaggtcgacggatc**agttgctattaacggtttcgg** | *S. bacillaris* CLIB3147 |
|  | H1-TDH3-Sb1kb-Rev | attaaaccagcaccgtcacc**gtccttcttggcaatcaaga** |  |
|  | HR-ForSb | aacgagttcggttactctgcccgtgttgttgacctccttgtcttgattgccaagaaggac**ggtgacggtgctggtttaat** | pFA6-GFP-hphMX |
|  | HR-RevSb | agaatcaactacttagagttactacttaaaagttggacaacatagatatgttcaaactgc**tcgatgaattcgagctcgtt** |  |
|  | H2-TDH3-Sb1kb-For | aacgagctcgaattcatcga**gcagtttgaacatatctatgttgtcc** | *S. bacillaris* CLIB3147 |
|  | H2-TDH3-Sb1kb-Rev | cactagtggatctgatatca**tttgtgacctcgtcggctcc** |  |
| **pFA6-TDH3.1kb.Td-GFP-Nat** | pFA6-TDH3-Td1kb-For | ccaatgaagcataagaataa**tgatatcagatccactagtggcct** | pFA6-GFP-NATMX |
|  | pFA6-TDH3-Td1kb-Rev | ccgaaaccgttaatagcaat**gatccgtcgacctgcagcgt** |  |
|  | H1-TDH3-Uni1kb-For | acgctgcaggtcgacggatc**agttgctattaacggtttcgg** | *T. delbrueckii* CLIB3069 |
|  | H1-TDH3-Td1kb-Rev | attaaaccagcaccgtcacc**agcagaggcaacgtgttcaa** |  |
|  | HR-ForTd | gacaacgaatacggttactctaccagagttgtcgacttggttgaacacgttgcctctgct**ggtgacggtgctggtttaat** | pFA6-GFP-NATMX |
|  | HR-RevTd | taaaacatgtcattaactaaaagtgaaaaaagcttgaaaactctttctcaactaattcat**tcgatgaattcgagctcgtt** |  |
|  | H2-TDH3-Td1kb-For | aacgagctcgaattcatcga**atgaattagttgagaaagagt** | *T. delbrueckii* CLIB3069 |
|  | H2-TDH3-Td1kb-Rev | cactagtggatctgatatca**ttattcttatgcttcattgga** |  |

**Bold nucleotides: sequence homologous to the template DNA**

**Supplementary Table 3: Primer list for cassette amplification**

| **Target species** | **Primer name** | **Sequence (5'→3')** |
| --- | --- | --- |
| ***S. cerevisiae*** | HR-ForSc | GACAACGAATACGGTTACTCTACCAGAGTTGTCGACTTGGTTGAACACGTTGCCAAGGCT**ggtgacggtgctggtttaat** |
|  | HR-RevSc | CTAAGTCATAAAGCTATAAAAAGAAAATTTATTTAAATGCAAGATTTAAAGTAAATTCAC**tcgatgaattcgagctcgtt** |
| ***L. thermotolerans*** | HR-ForLt | GACAACGAGTACGGTTACTCTACCAGAGTTGTCGACTTGGTTGAGCACGTCGCCAGCGCC**ggtgacggtgctggtttaat** |
|  | HR-RevLt | TATCAAGTCGTTAGATTTCGTTATAAAAAATGTCTTACGTAAAAATGTGTATCACGGAGT**tcgatgaattcgagctcgtt** |
| ***T. delbrueckii*** | K7-HR-TDH3.1kb-Td-For | **ATTGCTATTAACGGTTTCGGT** |
|  | K7-HR-TDH3.1kb-Td-Rev | **TTATTCTTATGCTTCATTGGAT** |
| ***S. bacillaris*** | K7-HR-TDH3.1kb-Sb-For | **GTTGGTATTAACGGTTTCGGTC** |
|  | K7-HR-TDH3.1kb-Sb-Rev | **TTTGTGACCTCGTCGGCTCC** |
| ***H. uvarum*** | K7-HR-TDH3.1kbHu-For | **GTTTCCATTAACGGTTTCGG** |
|  | K7-HR-TDH3.1kbHu-Rev | **ATATAAAAAGTTTAAGAAATAAAACGA** |

UPPERCASE: Sequence homologous to integration locus, **bold nucleotides: Sequence homologous to plasmid**

**Supplementary Table 4: Primer list and PCR conditions for checking the integration at the locus**

| **Target species** | **Primer name** | **Sequence (5'→3')** | **Primer pair** | **PCR cycle*** |
| --- | --- | --- | --- | --- |
| **Cassette - universal** | C | acagtcacatcatgcccctg |  |  |
|  | D | cacacctctaccggcagatc |  |  |
| ***S. cerevisiae*** | A-Hu | GGTATGGCTTTCAGAGTCCCA | A-Hu + C | 95°C – 5 min  [95 °C – 30 sec  55 °C – 30 sec  72 C – 1 min 30] x30  72°C – 5 min |
|  | B-Sc | TCAGAATCGTTATCCTGGCGG | B-Sc + D | 95°C – 5 min  [95 °C – 30 sec  50 °C – 30 sec  72 C – 2 min] x30  72°C – 5 min |
| ***L. thermotolerans*** | A-Hu | GGTATGGCTTTCAGAGTCCCA | A-Hu + C | 95°C – 5 min  [95 °C – 30 sec  55 °C – 30 sec  72 C – 1 min 30] x30  72°C – 5 min |
|  | B-Lt | GAGGAGGTGAAGGTGCAGAG | B-Lt + D | 95°C – 5 min  [95 °C – 30 sec  57 °C – 30 sec  72 C – 2 min] x30  72°C – 5 min |
| ***T. delbrueckii*** | A-Td-verif-1kb | TAGATGGTGTGCGTGCAATG | A-Td-verif-1kb + C | 95°C – 5 min  [95 °C – 30 sec  58 °C – 30 sec  72 C – 3 min] x30  72°C – 6 min |
|  | B-Td-verif-1kb | GCTGGCTACCGTTATTGTGG | B-Td-verif-1kb + D | 95°C – 5 min  [95 °C – 30 sec  58 °C – 30 sec  72 C – 3 min] x30  72°C – 6 min |
| ***S. bacillaris*** | A-Sb-verif-1kb | CACAAACTAACACCGTGGCA | A-Sb-verif-1kb + C | 95°C – 5 min  [95 °C – 30 sec  58 °C – 30 sec  72 C – 3 min] x30  72°C – 6 min |
|  | B-Sb-verif-1kb | TTGTGTTAGCCTGAAACCGC | B-Sb-verif-1kb + D | 95°C – 5 min  [95 °C – 30 sec  58 °C – 30 sec  72 C – 3 min] x30  72°C – 6 min |
| ***H. uvarum*** | A-Hu-verif-1kb | ATGACACACCACCCTCATGT | A-Hu-verif-1kb + C | 95°C – 5 min  [95 °C – 30 sec  58 °C – 30 sec  72 C – 3 min] x30  72°C – 6 min |
|  | B-Hu-verif-1kb | TGCTCTCGTGTACAGTCATTG | B-Hu-verif-1kb + D | 95°C – 5 min  [95 °C – 30 sec  60 °C – 30 sec  72 C – 3 min] x30  72°C – 6 min |
| **Cassette - universal** | C | acagtcacatcatgcccctg |  |  |
|  | D | cacacctctaccggcagatc |  |  |

* Polymerase used for all amplifications: Taq Polymerase (Thermo Fisher Scientific, Ref ep0402)


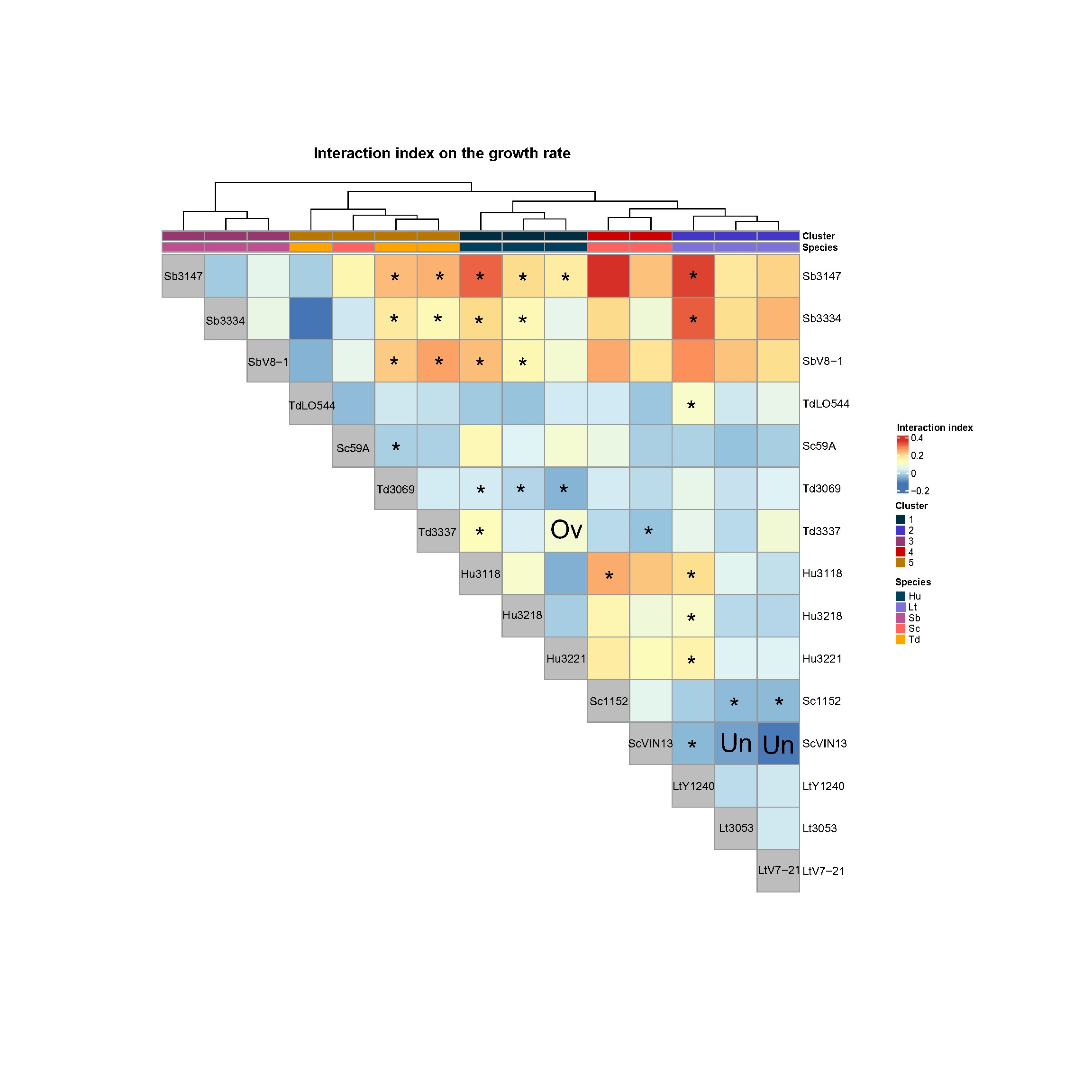


*Fig. S1: Heatmap of the growth rate (r): positive index means a higher growth rate so a positive interaction. * denotes cocultures whose growth rate is significantly different from the growth rate of both monocultures. Un denotes underyielding (latency time significantly longer than both monocultures), Ov denotes overyielding.*


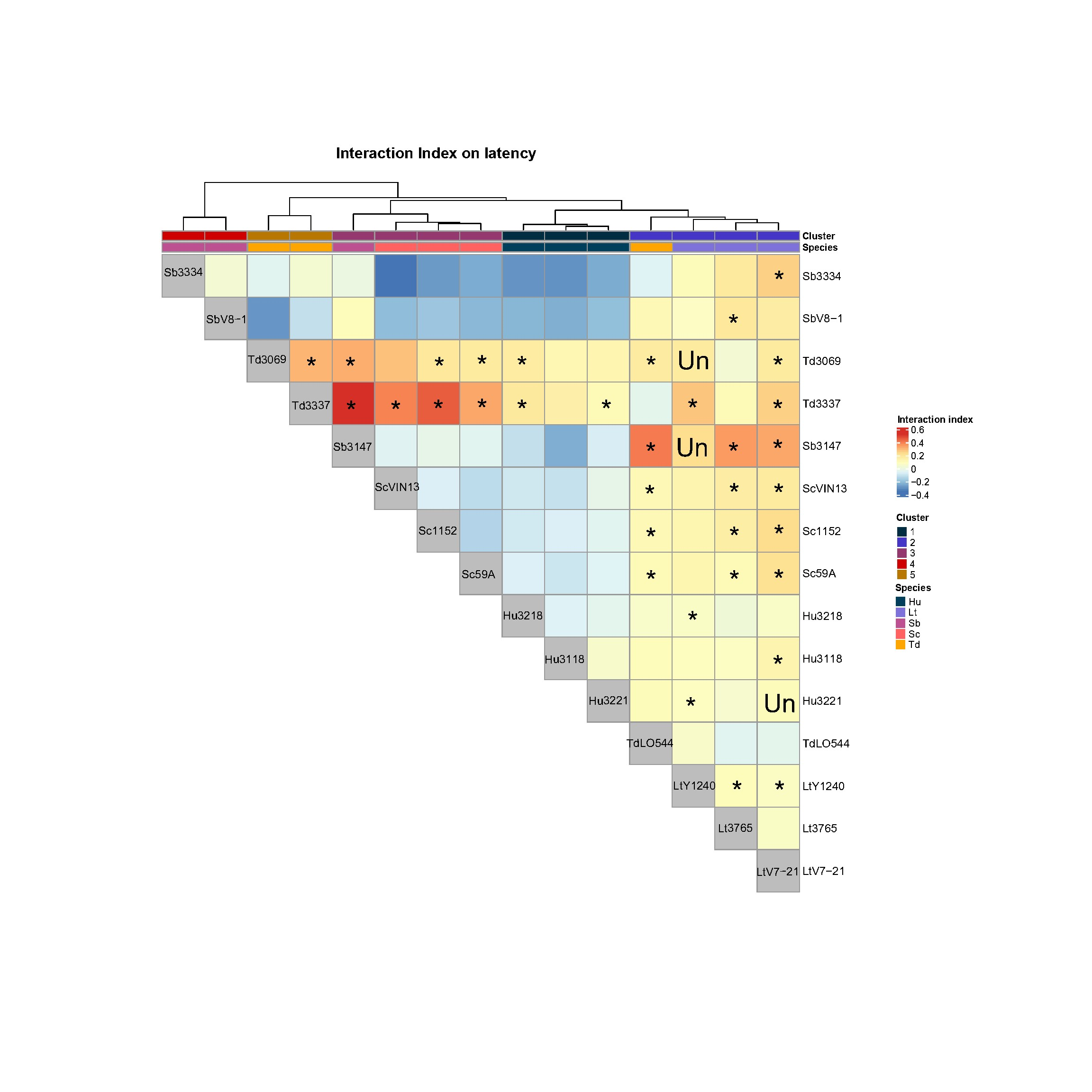


Fig. S2: *Heatmap of the latency index: positive index means a longer latency time so probably related to negative interactions. * denotes cocultures whose latency is significantly different from the average latency of both monocultures. Un denotes underyielding (latency time significantly longer than both monocultures). No overyielding was observed for the latency.*


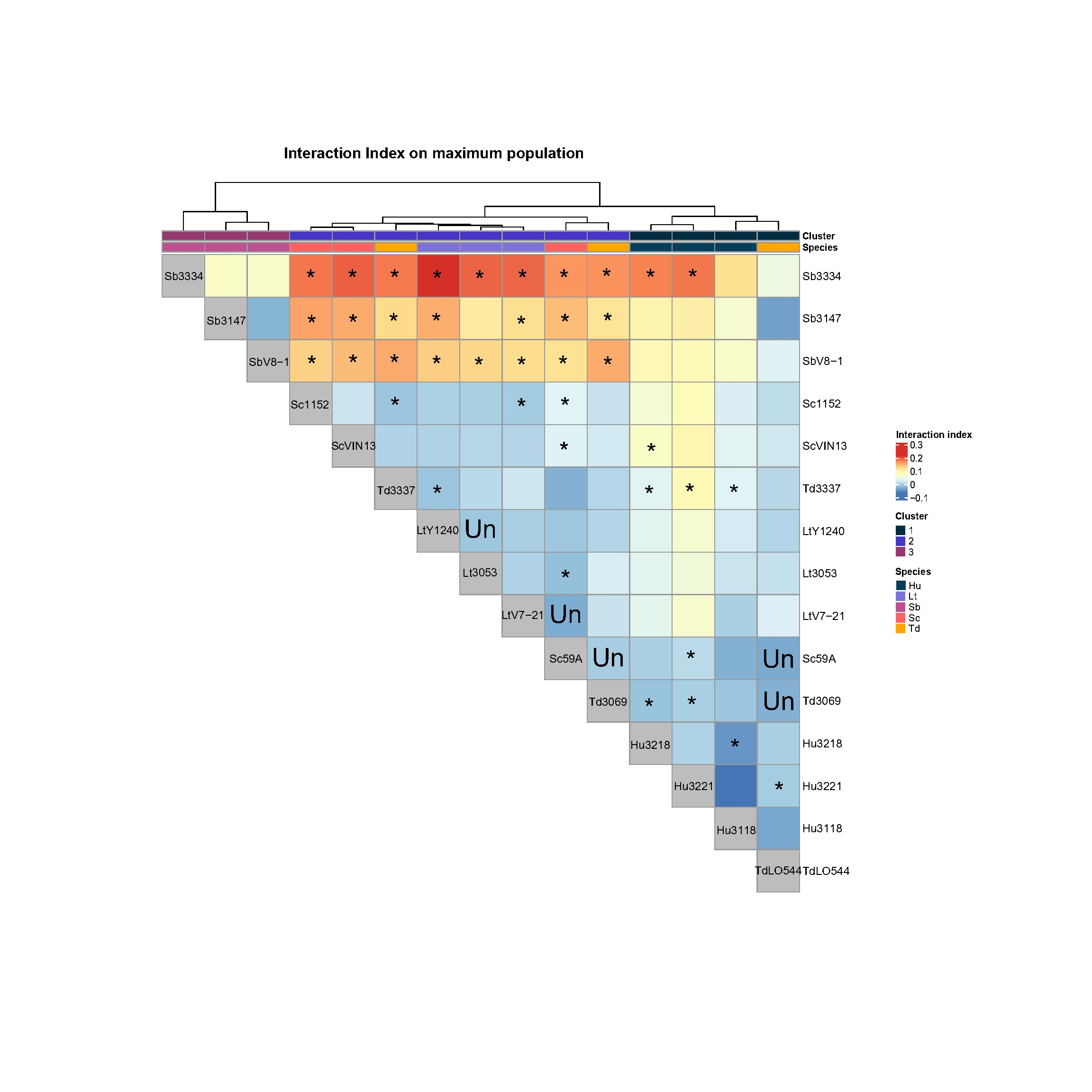


Fig. S3*: Heatmap of the maximum population (=MaxOD_600_): positive index means a higher maximum population so related to positive interactions. * denotes cocultures whose maximum population is significantly different from the average maximum population of both monocultures. Un denotes underyielding (latency time significantly longer than both monocultures)*
